# Segmentation and classification of skin lesions using hybrid deep learning method in the Internet of Medical Things

**DOI:** 10.1111/srt.13524

**Published:** 2023-11-15

**Authors:** Arslan Akram, Javed Rashid, Muhammad Arfan Jaffar, Muhammad Faheem, Riaz ul Amin

**Affiliations:** ^1^ Department of Computer Science and Information Technology Superior University Lahore Lahore Pakistan; ^2^ MLC Research Lab Okara Pakistan; ^3^ Information Technology Services University of Okara Okara Pakistan; ^4^ School of Technology and Innovations University of Vaasa Vaasa Finland; ^5^ Department of Computer Science University of Okara Okara Pakistan

**Keywords:** deep learning, Internet of Medical Things, ISIC‐2020, melanoma skin cancer, MRCNN, ResNet50

## Abstract

**Introduction:**

Particularly within the Internet of Medical Things (IoMT) context, skin lesion analysis is critical for precise diagnosis. To improve the accuracy and efficiency of skin lesion analysis, CAD systems play a crucial role. To segment and classify skin lesions from dermoscopy images, this study focuses on using hybrid deep learning techniques.

**Method:**

This research uses a hybrid deep learning model that combines two cutting‐edge approaches: Mask Region‐based Convolutional Neural Network (MRCNN) for semantic segmentation and ResNet50 for lesion detection. To pinpoint the precise location of a skin lesion, the MRCNN is used for border delineation. We amass a huge, annotated collection of dermoscopy images for thorough model training. The hybrid deep learning model to capture subtle representations of the images is trained from start to finish using this dataset.

**Results:**

The experimental results using dermoscopy images show that the suggested hybrid method outperforms the current state‐of‐the‐art methods. The model's capacity to segment lesions into distinct groups is demonstrated by a segmentation accuracy measurement of 95.49 percent. In addition, the classification of skin lesions shows great accuracy and dependability, which is a notable advancement over traditional methods. The model is put through its paces on the ISIC 2020 Challenge dataset, scoring a perfect 96.75% accuracy. Compared to current best practices in IoMT, segmentation and classification models perform exceptionally well.

**Conclusion:**

In conclusion, this paper's hybrid deep learning strategy is highly effective in skin lesion segmentation and classification. The results show that the model has the potential to improve diagnostic accuracy in the setting of IoMT, and it outperforms the current gold standards. The excellent results obtained on the ISIC 2020 Challenge dataset further confirm the viability and superiority of the suggested methodology for skin lesion analysis.

## 1 INTRODUCTION

Too much sun exposure increases your risk of developing melanoma, a form of skin cancer affecting individuals of all ages. Although it accounts for only 1% of all cases, it can quickly spread to other body parts if left untreated. The use of solariums and tanning beds has been linked to a 47% increase in the incidence of melanoma during the past decade, according to the statistics.^1^ In the United States, 196 060 new cases of melanoma were diagnosed in 2020, resulting in 6850 deaths. According to the World Health Organization (WHO), 9500 Americans are diagnosed with skin cancer daily.^2^ It is common practice for dermatologists and other medical experts to spot skin cancer simply by looking at the affected area. The dermatologist will thoroughly examine any moles, growths, or lesions that may be precursors to skin cancer. To give you the most complete analysis possible, they may employ dermatoscopes and other tools. The dermatologist looks at the skin lesions to determine their symmetry, border smoothness, color uniformity, size range, and pace of change. The letters ABCDE stand for “asymmetry, border irregularity, color variation, diameter, and evolution,” which together characterize these characteristics. While these signs can aid in a diagnosis, nothing beats the dermatologist's training and expertise in skin cancer. Chemiluminescence microscopy, or dermoscopy, is another common method.^3^

Connected medical equipment and software that share data over the Internet are collectively known as the “Internet of Medical Things” (IoMT).^4^ Wearable fitness trackers, remote patient monitoring systems, smart medical implants, and healthcare‐related smartphone apps all fall under this technology category. The IoMT has been a game‐changer in the medical field by supplying real‐time patient data and empowering doctors to provide better care and see better patient results.^5^, ^6^, ^7^ This technology is essential in today's healthcare systems because of its ability to expand patient access to care, lower hospitalization rates, and increase the prevalence of preventative medicine through constant monitoring.

With the help of IoMT, medical equipment, wearable sensors, and healthcare networks can all work together to keep tabs on their patients around the clock.^8^ IoMT sensors and devices can provide real‐time information on a wide range of skin characteristics and lesions, making them suitable for early cancer detection. This ongoing flow of information can teach deep learning algorithms to spot even the smallest changes in skin lesions, allowing for more rapid and accurate diagnosis.^9^ This facilitates earlier detection of skin malignancies and equips individuals to take charge of their health through regular, noninvasive examinations. In addition, IoMT paves the door for telemedicine, which allows patients to consult with dermatologists remotely, increases the number of readily available specialists, and guarantees that patients receive treatment quickly.^10^ With IoMT and deep learning, skin cancer detection could be greatly enhanced, allowing for earlier diagnosis and treatment for many more people.^11^, ^12^

In dermoscopy, a tiny region of skin is magnified and illuminated with the help of handheld equipment called a dermatoscopy. This procedure makes better observation and identification of the pigment network, vascular patterns, and other structures associated with skin cancer possible. Reducing the amount of difference between samples is possible through the use of computer‐based classification.^13^ Computer‐aided dermatological image categorization methods have been evolving to address data deficiency and imaging complexity. Dermoscopy is used to collect photographs of the skin, while a biopsy and a microscope are required to obtain images of other medical structures.^14^ State‐of‐the‐art methods for skin image classification entailed extensive preprocessing, segmentation, and feature extraction. Extensive work in medical image processing has focused on skin cancer categorization to facilitate early identification and better precise diagnosis. The difficulty of differentiating malignant from benign skin lesions has prompted the development of several cutting‐edge techniques. Convolutional Neural Networks (CNNs)^15^, ^16^ and other deep learning algorithms^17^, ^18^, ^19^, ^20^, ^21^, ^22^ have shown great progress in various skin cancer classifications, with excellent accuracy and robustness. Extraction of discriminative features from photos of skin lesions has been used to obtain outstanding classification results using DenseNet,^23^ InceptionNet,^24^ and ResNet.^25^ In addition, ensemble learning approaches have been used to improve classification performance by combining the strengths of numerous classifiers into a single model. These techniques have already proven their worth in dealing with the complexity and variability of skin lesion images.

Accurate skin lesion segmentation and classification approaches to enhance skin cancer diagnosis are the focus of this study. While deep learning has made great strides, it still has a long way to go before it can reliably segment skin lesions and achieve high classification accuracy. Artifacts such as hairs and other undesired features in the photos may cause existing approaches to perform poorly throughout the segmentation process. In addition, the discriminative features included in the segmented regions may need to be utilized more by the classification models, resulting in poorer classification accuracy.

Therefore, the purpose of this study is to present a mask‐region CNN hybrid approach to accurate skin lesion segmentation, with a special emphasis on the global thresholding technique for getting rid of unwanted hairs and aberrations. In addition, a transfer learning technique involving the Residual Neural Network (ResNet) is employed to guarantee correct classification. The proposed method is superior to previous state‐of‐the‐art methodologies in classifying skin lesions. This research addresses these concerns and contributes to the improvement of skin cancer diagnostic methods.

The main contribution of this research is described as follows:

- This research introduces an accurate and automated skin lesion segmentation technique that effectively removes unwanted artifacts, including hair, using MRCNN.
- The study employs the ResNet model to classify skin lesions, utilizing a transfer learning approach to achieve highly accurate classification results.
- The research provides a comprehensive comparison of the proposed methodology with existing techniques for segmenting and classifying skin lesions, highlighting the advancements and benefits of the novel approach.

Here is the rest of the paper: The literature review can be found in Section 2. The third section explains the materials and procedures used. Experiment details, together with analysis and commentary, can be found in Section 4. Sections 5 and 6 represent the summary and the research needs, respectively.

## 2 LITERATURE REVIEW

One form of skin cancer that causes malignant tumors on the skin is melanoma. Photographs taken using a dermatological lens can cause skin cancer. Using machine learning to identify skin cancer with high classification accuracy is based on high‐performance images. Texture analysis with the GLCM (Gray Level Co‐occurrence Matrix) was applied by Sheha et al.^13^ on photos from 2016 and medical clinics to automatically distinguish between melanocytic nevus and malignant melanoma. A Fisher score ranking was used to choose the 12 most important characteristics in the two classification strategies. Classification 1 used automatic multilayer perceptron (AMLP), which correctly identified skin cancer 76% of the time during testing; Classification 2 used multilayer perceptron (MLP), which was successful 92% of the time. For melanoma skin cancer identification on skin cancer photos from the dataset, Garg et al.^1^ presented the ABCD rule technique, which evaluated the skin lesion on different parameters, including asymmetry, border irregularity, color, and diameter. Before segmenting the image using Otsu thresholding, the pixel values of the cancerous zone were modified with MATLAB tools such as IMFILTER, IMADJUST, and the median filter. The cancer lesions were then classified with 91.6% accuracy using an abbreviated version of the ABCD rule, where each letter was given a different weight.

Ioannis Giotis et al.^26^ presented the decision system MED NODE, which uses color and textural information on dermoscopy images of skin cancer. The lesions were divided into two groups using k‐means clustering. Visual diagnostic characteristics, color features, and color‐textual features were used to classify segmented lesions with a 59% accuracy rate utilizing Color Image Analysis Learning Vector Quantization (CIA‐LVQ), Cluster‐based Adaptive Metric (CLAM), and color features. Taufiq et al.^27^ developed the m‐skin app for smartphones to aid in the detection of melanoma skin cancer at an early stage. The used skin photos were generously donated by the Clinic and Poliklinik for Dermatology and Allergology at Germany's Technische Universitat Munchen. After utilizing the Grab Cut method^24^ to segment the skin cancer lesions, an SVM classifier correctly identified 80% of the cases. Using standard classifiers and manually crafted features, Hardie et al.^28^ successfully segmented malignant regions with support vector machine (SVM) regression, achieving an accuracy of 70.10%. An SVM classifier labeled the lesions with 200 manually generated features, five‐fold cross‐validation, and validation image recall.

A study presented an automated skin cancer classification method using a Residual Deep Convolutional Neural Network (RDCNN) for the most frequent type. RDCNN was painstakingly trained and tested using six skin cancer datasets: PH2, DermIS, Quest, MED‐NODE, ISIC2016, ISIC2017, and ISIC2018, with three experiments. First, unfiltered dataset photos were tested; second, segmented images. A model from the second trial was retrained with a different dataset in the third. The proposed RDCNN outperformed previous deep convolutional networks in skin lesion categorization.^29^ As computer‐aided skin lesion diagnosis methods gained popularity, the second study thoroughly analyzed 53 classical machine learning and 49 deep learning studies from reliable databases over the previous 5 years to assess their accuracy. This review examined skin lesion segmentation and classification problems such as limited datasets, ad hoc picture selection, and potential racial biases by comparing contributions, methodologies, and outcomes.^30^ Finally, the authors introduced a novel deep CNN‐based method. This method segmented the region of interest, augmented ROI photos, and used deep convolutional network architectures, including AlexNet, ResNet101, and GoogleNet, for lesion identification in skin color photographs. The modified GoogleNet achieved 99.29, 99.15, and 98.14% classification accuracies for MED‐NODE, DermIS & DermQuest, and ISIC 2017 datasets, respectively.^31^

The writers discuss the extremely important topic of melanoma early diagnosis. Detecting melanoma at an early stage greatly increases the likelihood of survival. The Scientistsʼ deep transfer learning model, which employs MobileNetV2, a robust CNN, to classify skin lesions as malignant or benign, is an attempt to overcome this challenge. Class imbalance exists in the ISIC 2020 dataset since there are so few malignant samples (less than 2%). To remedy this situation and broaden the scope of the dataset, data augmentation techniques are employed. In order to improve skin cancer diagnosis, the suggested deep learning algorithm has been shown to surpass state‐of‐the‐art accuracy and compute efficiency techniques in experimental settings.^18^

Using images from the ISIC‐2017 and PH2 datasets,^32^ Xie et al.^33^ suggested a Mutual Bootstrapping Deep Convolutional Neural Networks (MB‐DCNN) model for simultaneously segmenting and classifying skin lesions. The model comprised an improved classification network, a mask classification network, and a rough segmentation network. In the ISIC‐2017 and PH2 datasets, the proposed approach shows an accuracy of 93.8%. For the ISIC 2019 dataset, Zhuang et al.^34^ created a cost‐sensitive multiclassifier active fusion framework, CS‐AF, employing 12 different CNN architectures. Classifiers trained in the Pytorch framework for as many as 40 iterations at a 103‐learning rate and 0.9‐momenta each achieved a substantial vote accuracy of 77.47%. Zhang et al.^35^ suggested a method for classifying skin lesions on the ISBI 2016 and ISBI 2017 datasets using convolutional networks (FCN) and shallow encoding networks with proprietary Textron features. The JACCARD Index was 0.8277 on ISBI 2016 and 0.7294 on ISBI 2017 using mini‐batch stochastic gradient descent (SGD) using the VGG16 model as the basis, velocity of 0.9, batch size of 20, rate when compared of 0.0001 that decreases to 0.001, and a dropout rate of 0.5. Using pictures from the ISBI 2016, ISIC 2017, and ISBI 2018, PH2, and HAM10000 datasets, Khan et al.^36^ proposed an automated technique for segmenting and classifying multiclass lesions based on deep discriminant characteristics. Lesion segmentation in 512 × 512 × 3 pictures was accomplished using 10‐layer CNNs. The segmentation accuracy on the ISBI 2016 dataset was 95.38%; on the ISBI 2017 dataset, it was 95.79%; on the ISIC 2018 dataset, it was 92.69%; and on the PH2 dataset, it was 98.70%. The segmented lesion was identified with a KELM accuracy rating of 90.67%.

Recent studies significantly focused on classifying skin cancer using various ML and DL techniques. The main problems in recent skin cancer detection are discussed. Images available for analysis are of different sizes due to the different shapes and sizes of lesions, leading to a lack of performance in the detection phase. In this regard, a preprocessing stage is required in the detection phase. Every human has a different skin structure. Some people have hair on their skin, some are without hair. In this sense, the image signal is corrupted. There should be a mechanism to remove hairs and other artifacts from skin images. Low contrast from nearby tissues might occasionally present extra challenges and make it more difficult to diagnose skin cancer correctly. Color lighting also creates specific challenges with components like color texture, light beams, and reflections. The human body has certain moles that may never turn into cancer cells, making it more challenging to reliably identify skin cancer from malignant photos. Another issue with detecting skin cancer is the current bias, which alters the performance of the algorithms to provide a better result. Table 1 shows the existing studies on skin cancer research.

**TABLE 1:** Comparison of skin cancer methods.

Reference	Methodology	Dataset	Classification accuracy
Garg et al.^1^	ABCD Rule Technique	ISIC	91.6%
Sheha et al.^13^	GLCM and Fisher Ranking	Self‐Collected	AMLP (76%) and MLP (92%)
Ioannis Giotis et al.^26^	MED NODE with CIA‐LVQ, CLAM	Dermoscopy Images	59%
Taufiq et al.^27^	m‐skin App with SVM	Skin Photos	80%
Hardie et al.^28^	SVM Regression	ISIC‐2018	70.10%
Xie et al.^33^	Mutual Bootstrapping DCNN	ISIC‐2017 and PH2	93.8%
Zhuang et al.^34^	CS‐AF with 12 CNNs	ISIC 2019	77.47%
Zhang et al.^35^	FCN and Shallow Encoding Networks	ISBI 2016 and 2017	JACCARD Index (0.8277 and 0.7294)
Khan et al.^36^	Deep Discriminant Characteristics	ISBI 2016, ISIC 2017, ISBI 2018, PH2	Segmentation accuracy (92.69–98.70%)
Anandaraj et al.^37^	Internet of Medical Things (IoMT) and cloud‐based skin lesion detection and classification model	ISIC	95.68%
Xiao et al.^38^	Few‐shot Prototype Network based on IoMT	mini‐ISIC‐2, mini‐ImageNet	78.34

## 3 MATERIAL AND METHODS

In this part, we detail the skin cancer detection algorithms, tools, and technology used in this investigation to identify melanomas. There were three main phases to accurately detect melanoma skin cancer: preprocessing, segmentation, and classification. The Mask R‐CNN (MRCNN)^39^ and ResNet^40^ hybrid model greatly enhances medical imaging skin lesion categorization. MRCNN and ResNet help this architecture segment and classify lesions. Mask R‐CNN is the latest deep‐learning model for image segmentation. Pixel‐level lesion masks determine object boundaries. RPN and FPN^41^ efficiently produce region proposals and capture multiscale features for MRCNN.

### 3.1 Proposed method

**FIGURE 1:**
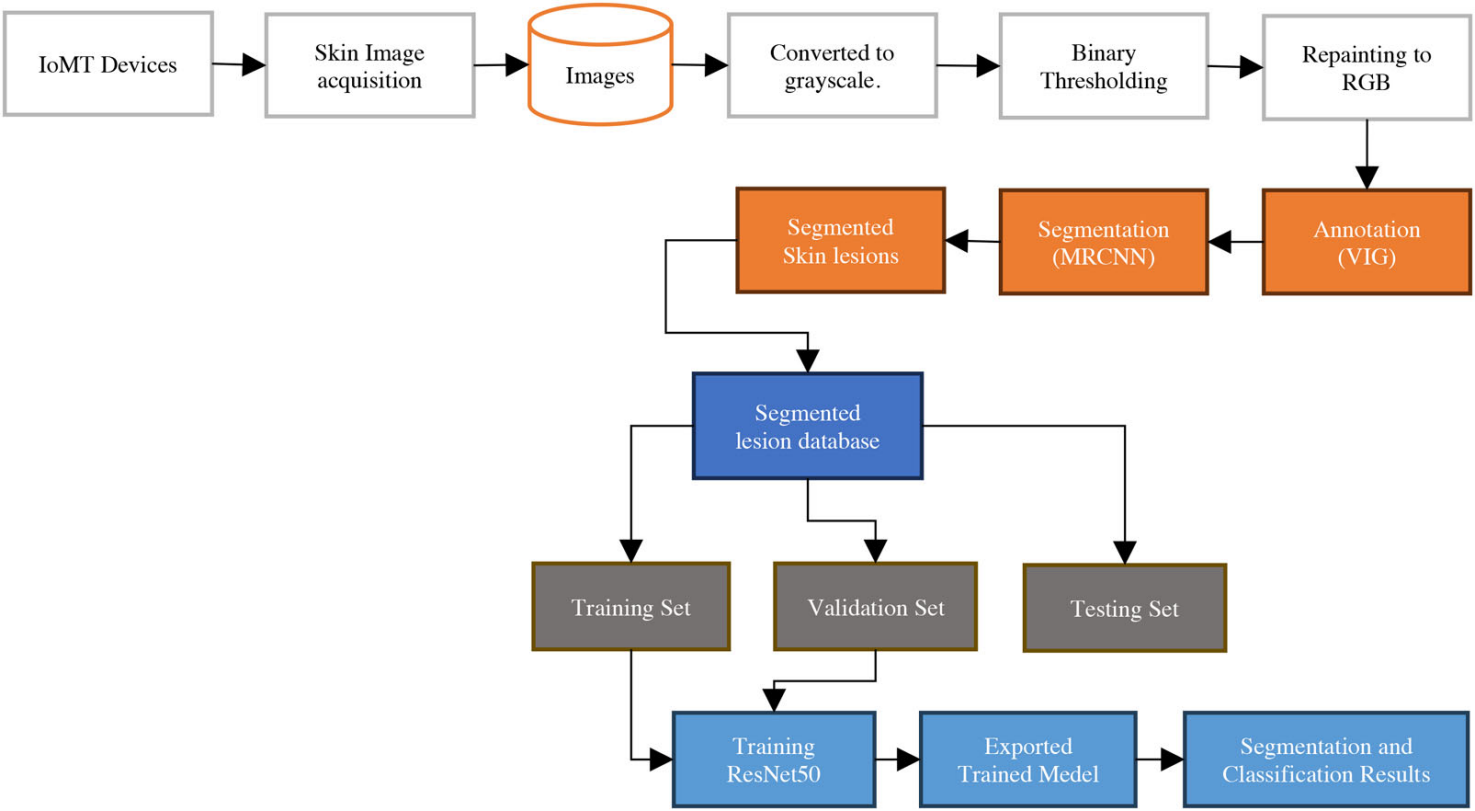
Proposed hybrid deep learning approach for segmentation and classification.

The proposed method for classifying skin lesions can be broken down into several phases. To begin, photos are taken from IoMT gadgets to create an image database for classifier training. This study's primary contribution is the development of thresholding techniques for improving recorded images, which eliminate hairs and other artifacts for improved understanding of skin lesions. Second, skin lesion classification is aided by locating and segmenting the lesions. Last, ResNet can train deep networks without causing the gradients to evaporate or become artificially inflated. ResNet can learn complex properties and patterns from data thanks to its use of skip connections and residual blocks. ResNet and MRCNN have been combined in this model. The hybrid network is powered by ResNet. Images of skin lesions exhibit advanced ResNet features. These characteristics are used by the MRCNN's central instance segmentation and bounding box regression. The head of MRCNN tweaks ResNet feature maps to focus on areas with lesions. To improve classification precision, MRCNN segmentation masks provide damage at the pixel level. Skin lesions can be categorized by the results of MRCNN and ResNet. A classifier receives features from the segmented regions and the ResNet backbone. Any classifier designed for a particular purpose could be utilized. The hybrid models segment the instances with an MRCNN and learn the features with a ResNet. Accurate segmentation and classification of skin lesions will aid dermatologists and other physicians in diagnosing and treating skin issues Figure 1 demonstrates proposed hybrid deep learning approach for skin lesion segmentation and classification.

#### 3.1.1 Dataset

This study uses dermoscopy skin lesion images from the ISIC Challenge. Images of skin lesions are included in the ISIC 2020^42^ challenge dataset for statistical evaluation. This dataset was produced to investigate and improve computer‐aided diagnosis systems for diagnosing and classifying skin illnesses, including the deadly melanoma. Dermatologists labeled photos in the dataset. These labels train and test machine learning algorithms to detect skin diseases. Wide range: The diverse ISIC 2020 Challenge dataset includes thousands of pictures from different people, geographic places, and clinical circumstances. Due to its diverse data formats, models trained on this dataset will generalize effectively. CAD for dermatological diagnoses relied on the ISIC 2020 Challenge dataset. It has allowed researchers to develop and test cutting‐edge algorithms for early skin condition detection and diagnosis, improving therapy and saving lives. Each picture received processing during the preprocessing phase.

#### 3.1.2 Data splitting

The ISIC Challenge skin lesion images were used for this analysis. The first step in the procedure involves processing each image. In the segmentation process, the images came from the training images collection. These photos were collected with obvious lesions to accurately segment the malignant zone. Only 10% of the data were used for testing and 90% for training. Test photos were used to evaluate the model. Table 2 provides details of the dataset used for experiments.

**TABLE 2:** Details of dataset.

Class	Training	Testing	Total
Benign	1200	200	1400
Malignant	1205	200	1405
**Total**	**2405**	**400**	**2805**

This study used skin lesion photographs from the ISIC Challenge. Processing each image is the initial stage of the operation. The photos used in the segmentation procedure were taken from the dataset of training images. Photos were taken with visible lesions to precisely segment the malignant area. We put 90% of the data through our training and only 10% via our testing procedures. The model was examined via test shots.

#### 3.1.3 Preprocessing

**FIGURE 2:**
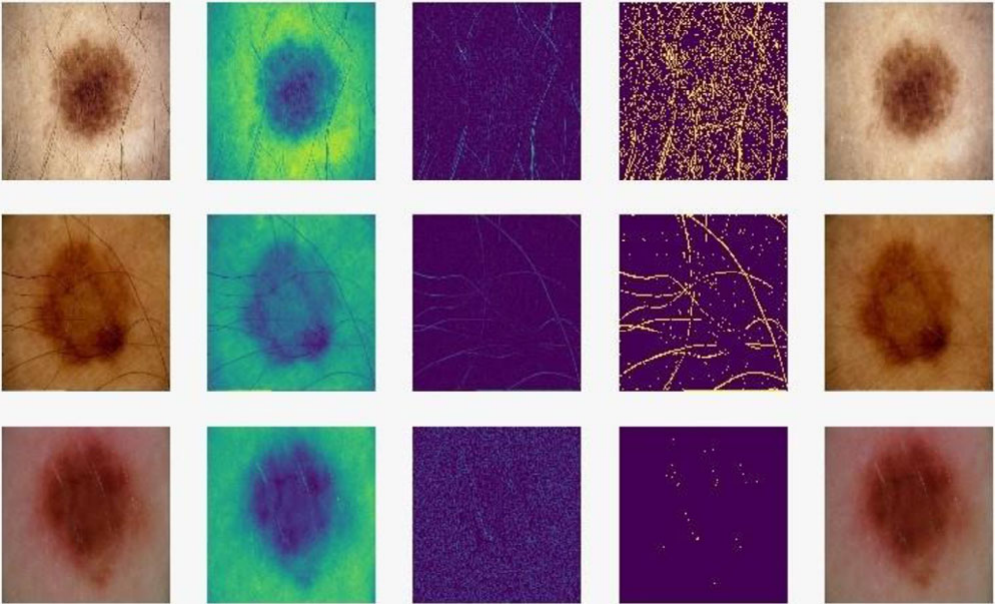
Pictures processed with BLACKHAT morphology are shown in Column 3, pictures processed with binary thresholding in Column 4, and images with unwanted hair eliminated in Column 5.

The photographs of skin cancer lesions feature hair, artifacts such as pen lines and rulers, and black frames. Since they can hinder melanoma detection, they should be removed to better ensure accurate skin cancer diagnoses. Several computer vision functions and approaches were employed to filter out the artifacts and hair, making detection easier. The following are steps for removing distractions from skin pictures. First, using the Python OpenCV package,^43^ the original photos were downscaled to 500 × 500 and turned to grayscale. Black Hat morphological filtering^44^ using cv2.MORPH BLACKHAT was used to identify the hair and artifact contours, with a 17 × 17 size kernel and one iteration. In subsequent steps, cv2 was used to do binary thresholding on the images. Use a THRESH BINARY with a value of 10 to emphasize the hair's natural shapes.^45^ A pixel with a value of 255 would be extremely brilliant, whereas a pixel with a value of 0 would be completely dark. The hair was painted out with a radius of 1 to clean up the original photos, and the finer details were filled in using cv2. INPAINT TELEA used the Fast March Method, which averaged the pixel weights of neighboring marked pixels, to fill in the blanks. As shown in Figure 2, a computer vision‐based technique was used to preprocess skin images by removing hairs and other artifacts.

#### 3.1.4 Segmentation

Backgrounds for skin lesions in images might vary widely. This meant that two different groups were affected by the lesion. The lesions were divided into smaller regions using instance segmentation with Mask RCNN (2017), which finds the contours of objects at the pixel level. The preprocessed pictures were processed in this step to remove the cancerous lesions of interest.

#### 3.1.5 Mask R‐CNN

**FIGURE 3:**
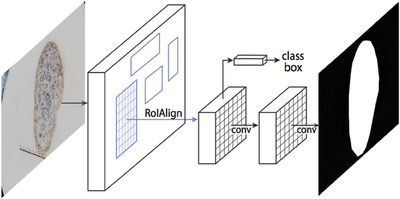
MRCNN architecture for skin lesion segmentation.

The deep neural network Mask Regional Convolutional Neural Network (MRCNN)^39^ solves instance segmentation problems in computer vision. Two additional convolutional layers generate a mask in the Faster RCNN variant. The first part analyzes the image and generates possible locations for the object. In the second stage, bounding boxes and masks are created to further classify the suggestions. The ROI alignment is used to digitize the boundaries of the cells and scale the targets to the same dimensions as the input. The values of the feature maps inside the cell are also calculated via interpolation. The architecture of the Mask RCNN is shown in Figure 3.

Mask R‐CNN introduces ROI Align. Pixel‐level segmentation outperforms ROI pooling because ROI aligns less spatial information. Bounding box regression adjusts the region recommendationsʼ bounding boxes until a better match is reached. Mask R‐CNN predicts pixel‐wise segmentation masks for each detected object using bounding boxes from object detection. The model estimates the box's object‐background pixel proportion. Mask R‐CNN's mask prediction module excels at instance segmentation, object identification, and semantic segmentation. Mask R‐CNN combines object identification with pixel‐level segmentation to accurately segment and recognize complex objects. It has contributed to computer vision for object detection, autonomous cars, and medical picture analysis.

#### 3.1.6 VGG IMAGE ANNOTATOR

**FIGURE 4:**
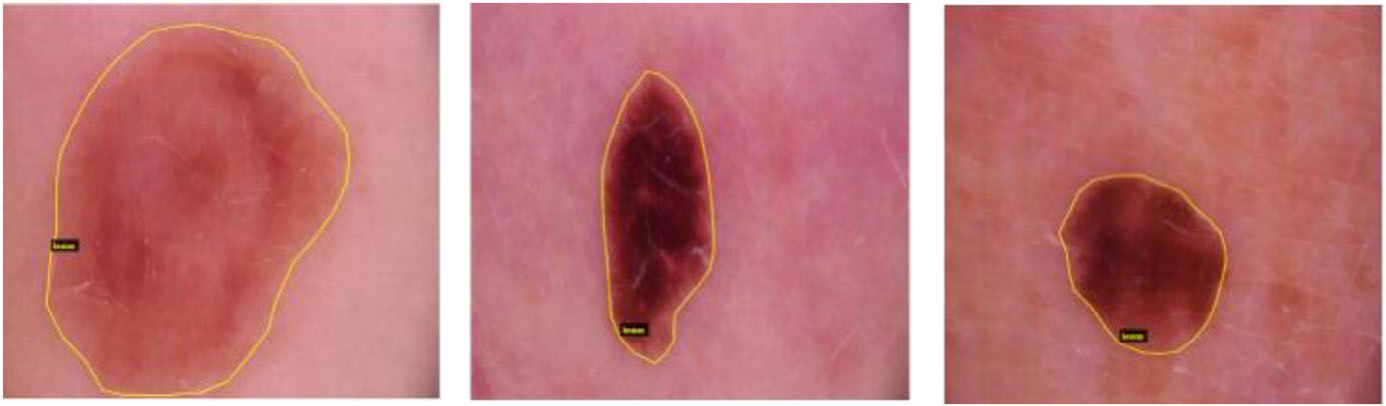
Annotated samples from the database using VGG Image Annotator.

The VGG Image Annotator^46^ generated a dataset using a coco‐like style. It is a straightforward method of manually annotating coco‐formatted images, videos, and audio files. The coco format dataset with segmentation masks for 80 classes was used to test mask RCNN. The photographs were all scaled down to 500px by 500px. The malignant zone and the noncancerous region were separated into the lesion and backdrop classes, respectively. Polygonal annotations indicating the lesion's presence can be observed in Figure 4.

Original images and JSON files containing polygons of each image will be used as input, and cropped images with lesions will be returned as output. The researchers in this study used the VGG Image Annotator to annotate the coco‐format training dataset, resulting in segmentation masks tailored to detect lesions. A thorough dataset description was written for this purpose, including its creation, distribution, and use.

#### 3.1.7 Classification

**FIGURE 5:**
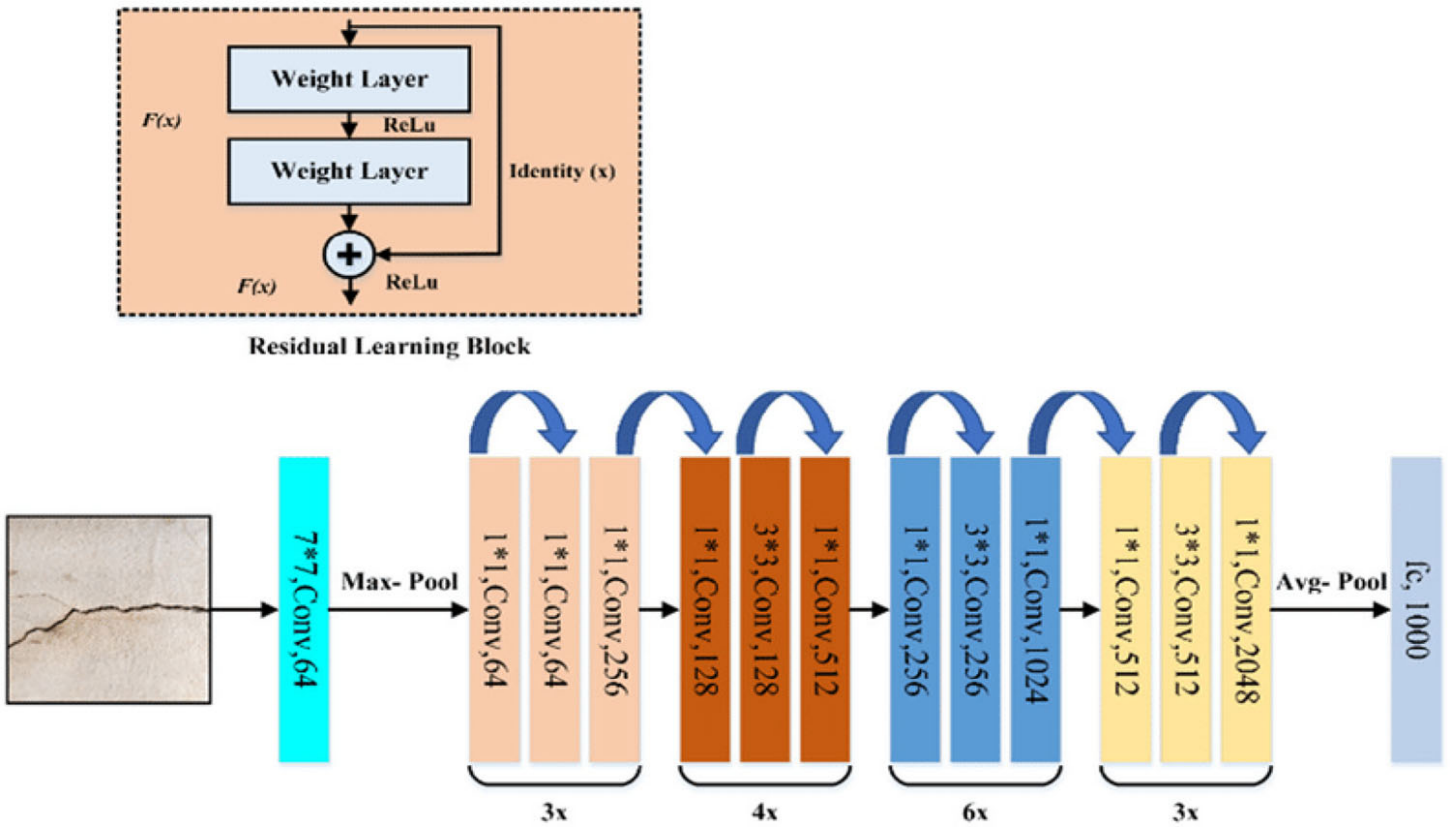
Resnet 50 architecture for skin lesion classification.

The segmented lesions were classified as malignant or benign using ResNet50, a CNN pretrained with weights from ImageNet. A 50‐layer ImageNet‐trained residual pretrained network was used in this study. The layer depth of this residual network is more than that of VGG networks, yet it is still simpler. Like other pretrained models, it was trained on the massive ImageNet dataset and could benefit from the improved depth and simplicity of optimization. A new CNN architecture called ResNet was developed to address the issue of disappearing gradients during very deep network training. Deep networks need help to learn complex mappings because their performance degrades as they become deeper. “Residual blocks” are ResNet's solution. ResNet learns residual mapping instead of the underlying mapping. “Shortcut connections” or “skip connections” allow information to travel from layer to layer and help the network learn the difference between input and output. ResNet's residual block is multilayered,^47^ mixing its output with the original input before passing on. In numbers,

(1) $$\;\;X_{out}=\;F\left(X_{in}+\;X_{in}\right)$$

where X_in It is the input to the residual block. F_in represents the transformation applied to the input by the layers within the block. X_out the residual block's output is the sum of the transformed input and the original input. By adding the input to the changed output, ResNet efficiently learns the residual mapping, allowing the network to focus on learning the “difficult” parts of the mapping rather than the complete mapping from scratch. This residual learning makes training incredibly deep networks with improved performance significantly easier. Figure 5 below depicts the multitiered architecture of ResNet. The model was built with the binary cross‐entropy loss function in mind, which allows predicting of two classes (benign and malignant). To implement the softmax^44^ in the last layer, the vector of integers was converted into a vector of probabilities using the following formula, where the probability for each value was proportional to the various scales of the column.

(2) $$softmax\;\left(Z_{i}\right)=\;\frac{\exp\left(Z_{i}\right)}{\sum_{j}\exp\left(Z_{i}\right)}$$

In this case, the value of the neurons in the output layer is denoted by the letter *Z*. Each picture is now 224 × 224 pixels in size. The network was built on top of a Resnet50 base with a dropout of 0.5 and batch normalization. We utilized a thick neuronal layer and a SoftMax activation function for binary classification. The Adam optimizer used binary cross entropy as its loss function. The model has been tweaked to run for 100 iterations, 50 steps, 64 batches, a base learning rate of 0.0001, and a minimum learning rate of 1e − 7 that is reduced by a factor of 0.2 if the validation accuracy fails after five iterations. We used Adaptive Moment Estimation (ADAM)^48^ with a fixed learning rate to train our segmentation and classification models. It uses the adaptive gradient method as a gradient‐based optimization strategy that yields stochastically optimal solutions (AdaGrad).

### 3.2 Experimental setup

TensorFlow and Keras will help Python implement the experiment. The pretrained ResNet50 model will be fine‐tuned with our skin lesion dataset to extract informative features from these photographs. Mask R‐CNN design in the classification pipeline allows accurate skin lesion segmentation. Mask R‐CNN's object recognition and instance segmentation capabilities allow us to generate pixel‐level masks for each lesion, improving the model's grasp of lesion boundaries. Transfer learning will initialize the ResNet50 spine of the integrated architecture with ImageNet weights. We will use a learning rate scheduler and momentum‐optimized SGD to fine‐tune the model. We will track accuracy, precision, and recall during training to evaluate the model. We will validate against a nontraining set sample to avoid overfitting and choose the best model. Grid and random search hyperparameter adjustment will optimize our model's settings. In Google Colab Pro,^49^ powerful GPUs will expedite training and inference. Google Colab Pro's session persistence will help us quickly refine the model and run many experiments.

### 3.3 Evaluation policy

A nonlinear classifier called ADAM is employed for classification. Since these are the most common metrics used in classification challenges, we looked at employing them to make our work competitive with other existing systems: True Positive Rate (TPR), True Negative Rate (TNR), and Area Under the Curve (AUC) (ROC). They are used to evaluate the efficacy of the method. Accuracy is the percentage of correctly identified samples relative to the total number of models. The accuracy metric is a wide‐ranging measure of a model's veracity. It considers both positive and negative samples that were appropriately classified as such. The formula takes the sum of all samples and divides it by the sum of all possible outcomes (both positive and negative).

(3) $$ACC\;=\frac{100\left(TP+TN\right)}{TP+TN+FN+FP}$$

The positive rate is calculated by counting the number of successfully identified samples. The TPR is the fraction of positive samples (true positives) that were properly recognized by the model relative to the total number of positive samples (true positives + false negatives).

(4) $$TPR\;=\frac{TP}{TP+FN}$$

The false positive rate is calculated using the percentage of incorrectly recognized samples. The FPR measures how often a model mistakenly classifies a sample as positive when it is negative. This metric is essential in areas where false positives might have serious effects, like medical diagnostics.

(5) $$FPR\;=\frac{FP}{FP+TN}$$

The precision ratio is the proportion of correctly classified samples relative to the total number of accurately predicted elements. Precision can be defined as the fraction of samples containing true positives relative to the total number of samples the model identifies as positive. It is a measurement that determines the accuracy of the model's positive predictions.

(6) $$Precision\;=\frac{TP}{TP+FP}$$

The recall is the proportion of adequately categorized samples to all correctly classified instances. The proportion of true positives among all actual positive cases, including those that are missed by the model and are referred to as false negatives, can be calculated using recall. In situations where it is very important to identify as many positive cases as possible, such as in medical diagnostics, it is highly useful.

(7) $$Recall\;=\frac{TP}{TP+FN}$$

The terms “T.P.,” “T.N.,” “F.P.,” and “F.N.” in these equations, respectively, stand for the number of true positives, true negatives, false positives, and false negatives. These metrics are used extensively in evaluating the performance of classification models. They balance various aspects of a model's effectiveness, depending on the particular context in which the problem is being addressed.

## 4 RESULTS AND DISCUSSION

In this section, we will use several metrics to assess how well the suggested method performs and compare that performance to that of established methods. I have broken down the study's findings and commentary into the following sections for your convenience.

4.1. Performance analysis of proposed MRCNN for segmentation of skin lesion.
4.2. Comparative analysis of proposed MRCNN with existing segmentation methods.
4.3. Performance analysis of proposed ResNet50 for classification of segmented skin lesion.
4.4. Comparative analysis of proposed ResNet50 with existing skin lesion classification methods.

### 4.1 Performance analysis of proposed MRCNN

MRCNN formed the basis of the model architecture, with 150 epochs, 100 steps, and 1 batch size as training parameters. The training was extremely efficient, with a momentum value of 0.9 and a base learning rate of 0.001. If there was no improvement in validation accuracy, the learning rate was decreased by 0.0001 to ensure effective learning. These methods train a model that can accurately segment and detect lesions using an annotated data set and hyperparameters customized specifically for MRCNN.

**FIGURE 6:**
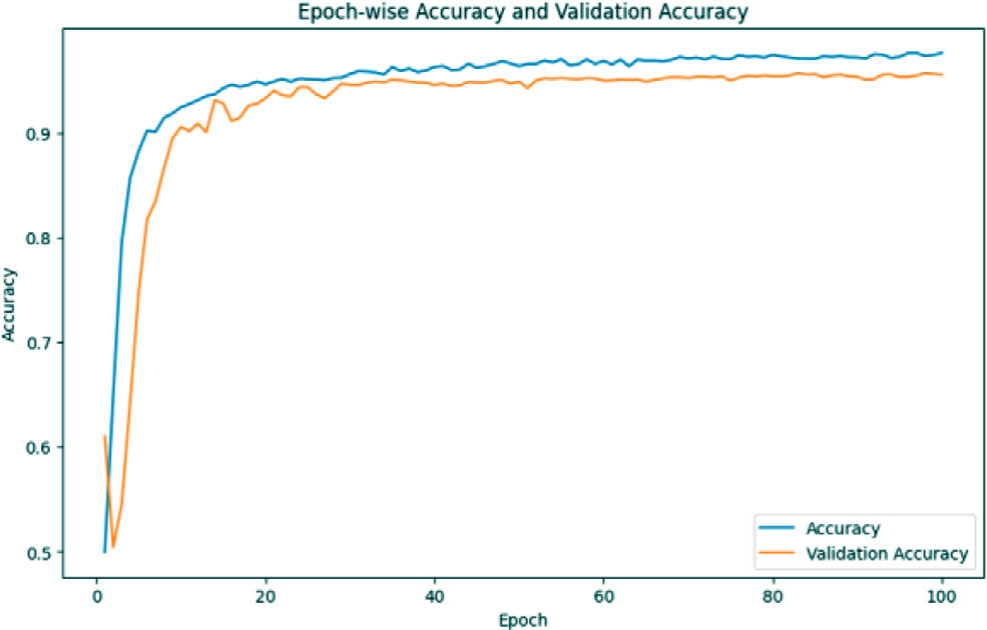
Training and validation accuracy graph of MRCNN.

The training accuracy is low at first (0.4997) but steadily improves as more iterations are performed. This indicates that the model is learning from the data it is fed. The accuracy of validations could be more consistent at first, but it improves over time. However, in most instances, validation accuracy is lower than training accuracy. Because the model typically slightly overfits the training data, this occurs frequently. As the training accuracy increases, so does the validation accuracy, providing evidence that the model is improving at generalization to new data. If accuracy improvements decrease to a trickle in later epochs (say, between Epochs 50 and 100), the model's performance may have plateaued. After 100 iterations, the model achieved a training accuracy of almost 97.14% and a validation accuracy of nearly 95.82%, both indicative of a job well done. Figure 6 depicts the training and validation accuracy graph of the segmentation model.

**FIGURE 7:**
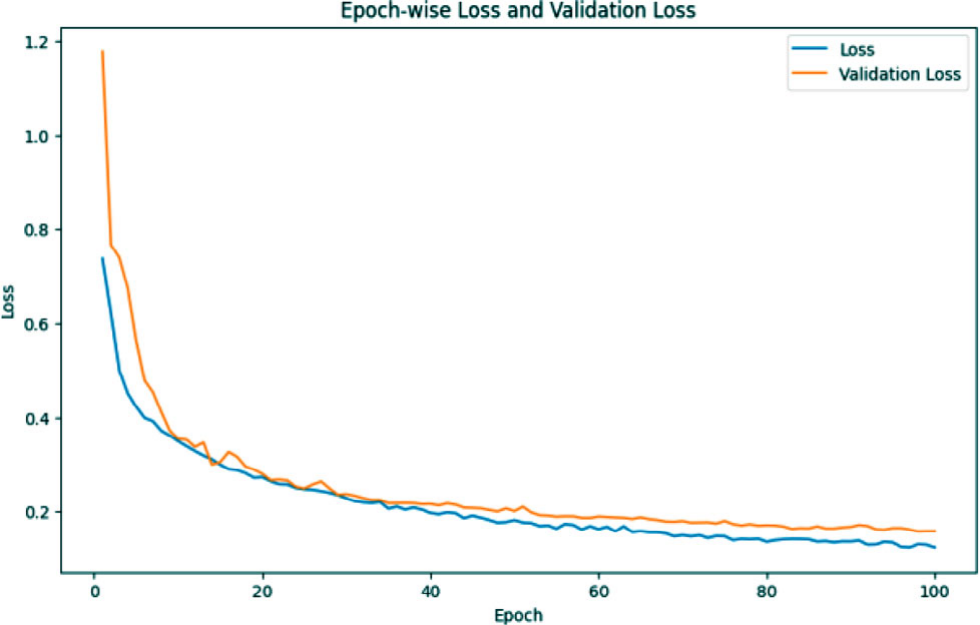
Training and validation loss graph of MRCNN.

It becomes clear that the model improves its fit to the training data as the training loss decreases. Additionally, the validation loss decreases as the model improves, showing that it can successfully generalize to new data. The training loss and the validation loss must be tracked closely when training. A decrease in training loss without a corresponding decrease in validation loss may indicate overfitting, in which the model memorizes the training data rather than learning the underlying patterns. High values for both training and validation losses suggest that the model is underfitting and does not faithfully represent the data. Figure 7 depicts the training and validation loss graph of the segmentation model.

**FIGURE 8:**
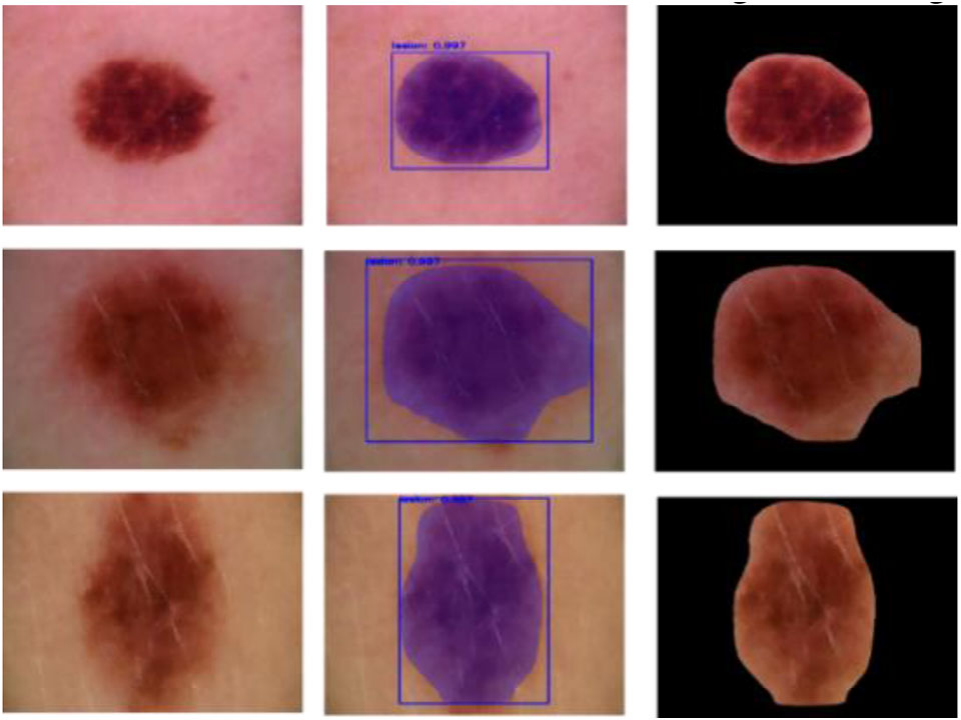
Results of segmentation using testing dataset (A) original images; (B) segmented masks created; (C) cropped images with lesion.

The results of the model's segmentation show how well it can find and separate items in photos. With 91.73% precision, the program can find the edges of objects. The model's recall rate of 94.17% shows that it can recognize many of the important things in the pictures. This exact segmentation gives an accuracy of 95.49%, which shows that the model is good at classifying pixels. The accuracy of the model's semantic picture segmentation is very good. But the 5.51 loss number may need to be looked at more closely since lower values are better. These results show how useful and effective the model is in semantic segmentation tasks, which can be used in medical imaging, self‐driving cars, and other places. Figure 8 depicts the testing results of the segmentation method.

Miss segmentation in skin lesion segmentation is caused by several factors, including the wide variety of lesion types, artifacts, the complexity of skin anatomy, uneven lesion borders, a lack of data, and poor algorithmic choices. In this study, skin images contain blurriness and have different types of skin lesions with almost the same color and structure as others, so misclassification occurs while training and testing the segmentation model.

### 4.2 Comparative analysis of proposed segmentation method with state‐of‐the‐art methods

Table 3 compares the proposed MRCNN skin lesion segmentation approach to numerous state‐of‐the‐art methods across datasets. The table has rows for methods and columns for citations, method names, datasets, and accuracy. High‐resolution CNNs boost accuracy to 93.80% on the ISIC‐2017 dataset (first row).^50^ Additionally, FCN^51^ uses fully convolutional networks to achieve 94.5% accuracy on the same ISIC‐2017 dataset. The boundary‐aware transformer (BAT) method is suggested for skin lesion segmentation.^52^ This method has 91.20% accuracy on ISIC‐2018. The next row describes a CNN‐based skin lesion segmentation algorithm using the ISIC‐2020 dataset that achieves 94.32% accuracy.^53^ The new Multi‐Scale Residual Encoding and Decoding network (MS‐RED)^54^ achieves 94.10% accuracy on the ISIC‐2017 dataset. In Liu et al.,^55^ NCR‐NET (an acronym for “Neighborhood Context Refinement Network”) obtains 94.01% accuracy on the ISIC‐2017 dataset. MSFNet, a Lightweight Multi‐Scale Feature Fusion Network, obtains 92.17% accuracy on the ISIC‐2018 dataset in the following column.^56^ Finally, Kaur and Ranade^57^ propose a CNN‐based skin lesion segmentation method that uses group normalization and a mixed loss function to achieve 91% accuracy on the ISIC‐2017 dataset. In the final column, MRCNN outperforms all competing approaches on the ISIC‐2020 dataset with 95.49% accuracy. Table 3 shows the results of testing multiple skin lesion segmentation methods on different datasets. Compare the proposed MRCNN method to state‐of‐the‐art methods; it more accurately segments skin lesions from dermoscopy images.

**TABLE 3:** Comparative analysis of proposed segmentation method with state‐of‐the‐art methods.

Citation	Method	Dataset	Accuracy
^50^, 2020	CNN	ISIC‐2017	93.80%
^34^, 2020	FCN	ISIC‐2017	94.58%
^35^, 2021	BAT	ISIC‐2018	91.20%
^36^, 2021	CNN	ISIC‐2020	94.32%
^37^, 2022	MS‐RED	ISIC‐2017	94.10%
^38^, 2022	NCR‐NET	ISIC‐2017	94.01%
^39^, 2023	MSFNet	ISIC‐2018	92.17%
^40^, 2023	CNN	ISIC‐2017	91%
**Proposed**	**MRCNN**	**ISIC‐2020**	**95.49%**

### 4.3 Performance analysis of proposed ResNet50

**FIGURE 9:**
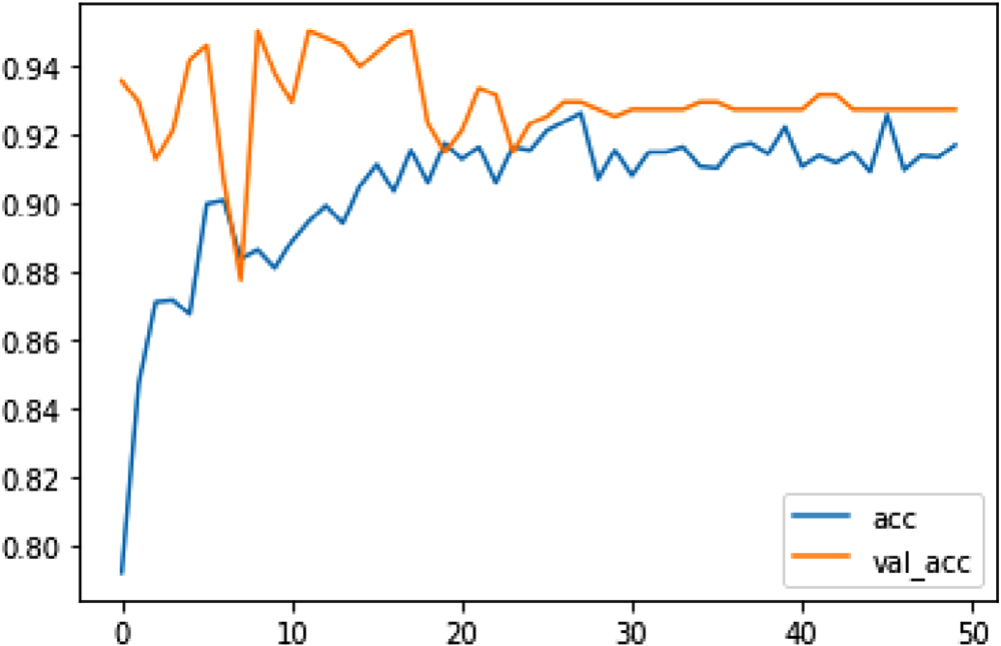
Training and validation accuracy graph of ResNet50.

The results of a deep learning model trained for 50 iterations are displayed in the output. The model is trained on one data set during an epoch, and its accuracy is measured using a different set of data known as validation data. After each epoch, the training and validation accuracy scores are recorded. The blue line depicts the model's success in acquiring knowledge from the training data, which stands for training accuracy. Beginning at about 79.5%, it improves with data‐driven model refinement. The training accuracy stabilizes at about 91% after 50 iterations. The red line indicates the validation accuracy, which measures how effectively the model extrapolates to new data. It begins at 93.56% and varies over the epochs. The precision of the validation fluctuates between 92 and 95%. Figure 9 depicts the training and validation accuracy graph of a classification model.

**FIGURE 10:**
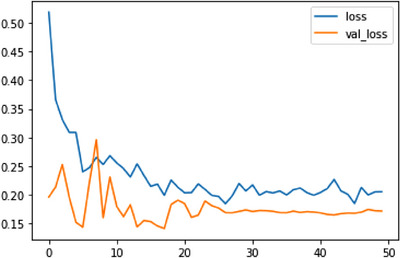
Training and validation loss graph of ResNet50.

The training and validation loss graphs for a deep learning model are shown. The blue line in this graph shows the training loss across 50 iterations, whereas the red line shows the validation loss. Epoch 1 sees a training loss of 53.58% and a validation loss of 19.60% for the model. As measured by a declining training loss, the model improves as it takes in more data. Validation loss decreases, indicating that the model becomes more accurate when applied to new data. Epoch 5 has a higher validation accuracy (val_acc) than Epoch 4, which was 93.56%. This indicates that the model is getting better at applying novel data. Validation accuracy reaches a maximum of 95.01% at Epoch 9, and the model continues to perform admirably through Epoch 13. This indicates the model's success on the validation dataset, where it achieved an accuracy of 95.01%. After Epoch 13, the model's performance on the validation data becomes slightly unstable, although it still has a very high level of accuracy. The training process is fine‐tuned by slowing the model's learning rate occasionally to avoid overshooting the optimal weights. It becomes apparent that the model is learning how to classify the skin lesions as the loss decreases and the validation accuracy increases across the training epochs. The model performs quite well, suggesting that it can correctly classify skin lesions without visual signals. Figure 10 depicts the training and validation loss graph of a classification model.

**FIGURE 11:**
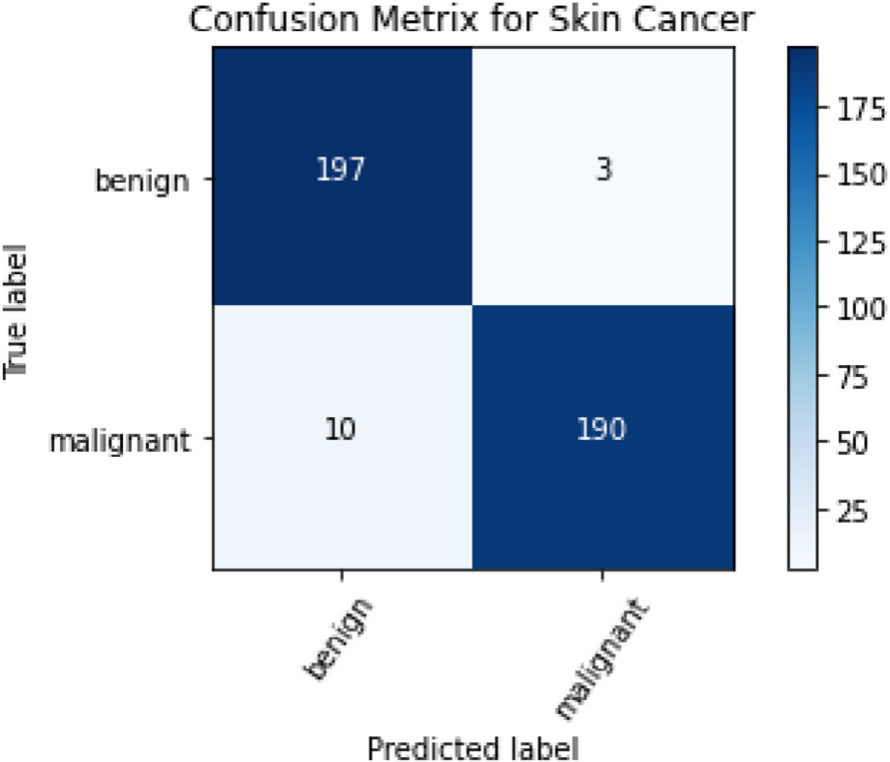
Confusion matrix for testing results using trained ResNet50.

The confusion matrix is needed to evaluate classification models on the test set. The report describes the model's predictions. This confusion matrix requires binary classification into “Benign” and “Malignant” classes. Finally, the confusion matrix helps evaluate the binary classification approach. The model's accuracy, precision, recall, and specificity are high. However, several indicators and analyses are needed to assess the model's performance. Figure 11 shows the confusion matrix of testing results from the trained classification model.

The findings show that a classification model can distinguish “Benign” from “Malignant.” It is estimated that the model is 96.75% accurate. With 91% precision, the model identifies “Benign” samples. The model has a 99% “Benign” class recall, meaning that it correctly identifies most “Benign” samples in the training dataset. This 95% F1 score for “benign” strikes a good mix between accuracy and recall. Precision for the “Malignant” class is 99%, indicating that when the model labels a sample as “Malignant,” it is usually correct 99% of the time. Since the model only has a 90% recall, it risks missing “Malignant” samples. The “malignant” F1 gets 94%. The testing results provide insights into distinguishing “Benign” from “Malignant” samples in the dataset, which provides a well‐performing model with excellent accuracy, precision, and recall for both classes. The efficacy of the model and its potential for improvement need to be measured using additional metrics and analysis. Table 4 represents the testing performance of the trained classification model.

**TABLE 4:** Testing results of the proposed Resnet50.

	Measures				
Class	Precision	Recall	F1	Acc	Avg. Acc
Benign	91%	99%	95%	98.5%	96.75
Malignant	99%	90%	94%	95%	

**FIGURE 12:**
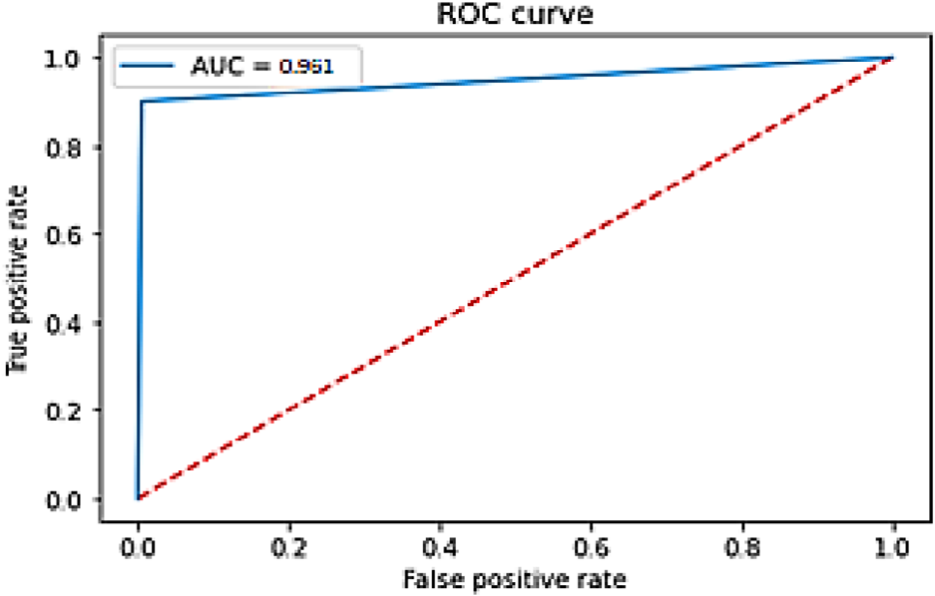
ROC curve for lesion class.

The ROC curve was also utilized to evaluate the precision of the model. As shown in Figure 12, the overall performance across all possible classification criteria was 96.1%, represented by the area under the ROC curve.

**FIGURE 13:**
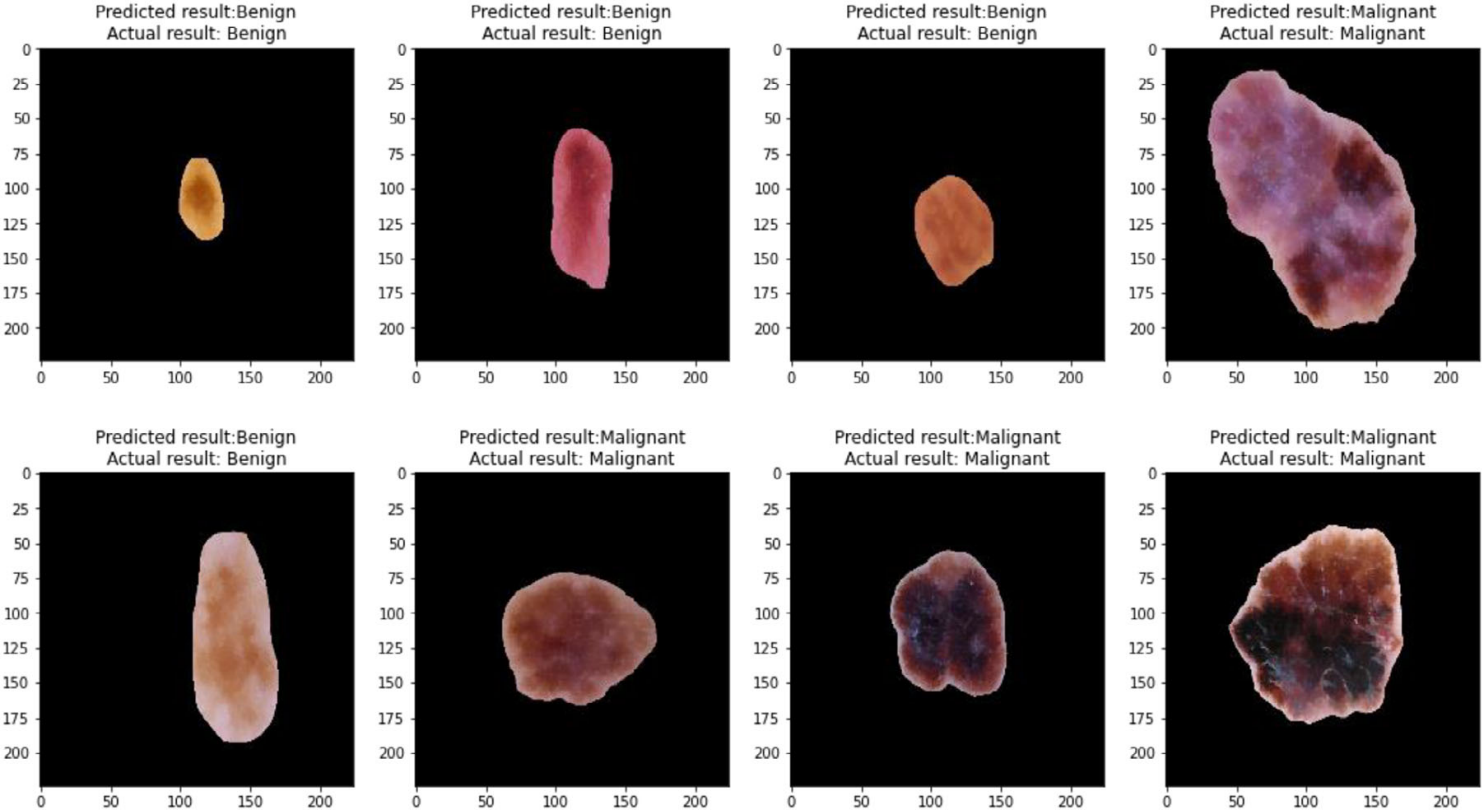
Results of lesion classification using ResNet50.

Figure 13 demonstrates a few melanoma detection findings. By visually contrasting the model's predictions with the data, the accuracy of a classification model can be demonstrated. Each image in the network represents a unique data instance, and specific pixel positions on the images correlate to the actual and anticipated class labels. By inspecting the graph, we may evaluate the accuracy of the model's predictions.

### 4.4 Comparative analysis of proposed ResNet50 with other state‐of‐the‐art methods

The proposed method for classifying skin lesions is compared to other, more advanced methods in Table 5. A methodology, reference, data collection, and precision are provided in each table entry. MB‐CNN, a mutual bootstrapping model for automatic skin lesion segmentation and classification, was proposed in 2020 by Xie et al.^18^ It achieved 93.8% accuracy on the ISIC‐2017 dataset. Second, in 2021, Khan et al.^20^ developed Deep CNN, which applied moth flame optimization with deep learning features to segment and classify skin lesions. It received 90.67% on the ISIC‐2017 dataset. In 2022, Gouda et al.^41^ proposed using deep learning and CNN to identify skin cancer from photographs of skin lesions. The accuracy on the ISIC‐2017 dataset was 83.20%. Finally, in 2023, DSNN (deep spiking neural network) was presented by Gilani et al.,^42^ which is a deep spiking neural network for skin cancer categorization. The accuracy on the ISIC‐2019 dataset was 89.57%. Hyperspectral image engineering for skin cancer classification was revolutionized by Huang et al.^43^ in 2023 with the release of YOLOv5. Unlike previous approaches, this one was validated on a dataset the authors developed, achieving an accuracy of 79.20%. Finally, on the ISIC‐2020 dataset, the greatest accuracy of the ResNet50‐based method was 96.75%. The ResNet50‐based method outperforms state‐of‐the‐art methods in the table, which compares methods used to classify skin lesions. A method's computational complexity and generalization capabilities should be considered for practical skin lesion categorization applications.

**TABLE 5:** Comparison of proposed classification method with state‐of‐the‐art methods.

Citation	Method	Dataset	Accuracy
^18^, 2020	MB‐CNN	ISIC‐2017	93.8%
^36^. 2021	Deep CNN	ISIC‐2017	90.67%
^41^, 2022	CNN	ISIC‐2017	83.20%
^42^, 2023	DSNN	ISIC‐2019	89.57%
^43^, 2023	YOLOv5	Self	79.20%
**Proposed**	**ResNet50**	**ISIC‐2020**	**96.75%**

## 5 CONCLUSION

A new era has begun for CAD systems, particularly in skin lesion analysis, thanks to the IoMT. This study introduces an innovative approach that successfully integrates semantic segmentation with lesion classification by employing a hybrid deep learning model. For precise semantic segmentation, the state‐of‐the‐art method employs an MRCNN architecture, whereas lesion categorization is handled by a trained ResNet50. Combining these techniques allows us to locate and map out the exact boundaries of lesions. A large, meticulously annotated set of dermoscopy images is used in the study to set the stage for efficient model training. The segmentation accuracy of the hybrid deep learning model is 95.49%, which is higher than the current state‐of‐the‐art approaches. Furthermore, contemporary methods of skin lesion classification represent significant advancements over its antecedents. In the ISIC 2020 Challenge dataset evaluation, the model performed exceptionally well, with an accuracy rate of 96.75%. Collectively, these results place the segmentation and classification models at the forefront of what is considered best practice in IoMT. This research paves the way for more precise skin lesion analysis and highlights IoMT's vast potential for enhancing medical diagnosis and therapy.

## 6 FUTURE WORK

The accuracy of the classification model can be improved in future studies if additional data are collected on the malignant kind. MRCNN was the foundation employed in the lesion segmentation process. Using the other pretrained model, omitting the Mask Layer, and relying on the Regional Proposal Network layer to acquire the bounding boxes of the interesting regions allows us to significantly shrink the model.

## CONFLICT OF INTEREST STATEMENT

The authors declare no conflicts of interest.

## CODE AVAILABILITY STATEMENT

The code will be available upon request to the corresponding author.

## Data Availability

The data will be available upon request to the corresponding author.

## References

[1] Garg N , Sharma V , Kaur P . Melanoma skin cancer detection using image processing. In: Sensors and Image Processing: Proceedings of CSI 2015 , Springer; 2018:111‐119.

[2] Mattiuzzi C , Lippi G . Cancer statistics: a comparison between world health organization (WHO) and global burden of disease (GBD). Eur J Public Health. 2020;30(5):1026‐1027. 31764976 10.1093/eurpub/ckz216

[3] Ferlay J , Colombet M , Soerjomataram I , et al. Cancer statistics for the year 2020: an overview. Int J Cancer. 2021;149(4):778‐789. 10.1002/ijc.33588 33818764

[4] Vishnu S , Ramson SRJ , Jegan R . Internet of Medical Things (IoMT)—an overview. In: 2020 5th International Conference on Devices, Circuits and Systems (ICDCS) . 2020:101–104. doi:10.1109/ICDCS48716.2020.243558

[5] Alarood AA , Faheem M , Al‐Khasawneh MA , Alzahrani AIA , Alshdadi AA , Secure medical image transmission using deep neural network in e‐health applications. Healthc Technol Lett. 2023;10(4):87‐98. 37529409 10.1049/htl2.12049 PMC10388229

[6] Gupta S , Sharma HK , Kapoor M . Integration of IoMT and blockchain in smart healthcare system. In: Gupta S , Sharma HK , Kapoor M , eds. Blockchain for Secure Healthcare Using Internet of Medical Things (IoMT). Springer International Publishing; 2023:79‐91. doi:10.1007/978-3-031-18896-1_7

[7] Ali G , Dastgir A , Iqbal MW , Anwar M , Faheem M . A Hybrid convolutional neural network model for automatic diabetic retinopathy classification from fundus images. IEEE J Transl Eng Health Med. 2023; 11:341‐350.

[8] Albahri AS , Alwan JK , Taha ZK , et al. IoT‐based telemedicine for disease prevention and health promotion: state‐of‐the‐art. J Netw Comput Appl. 2021;173:102873. doi:10.1016/j.jnca.2020.102873

[9] Khan AA , Madendran RK , Thirunavukkarasu U , Faheem M , D2PAM: epileptic seizures prediction using adversarial deep dual patch attention mechanism. CAAI Trans Intell Technol. 2023;8(3):755‐769.

[10] Gao J , Lyu C , Qiao X , Tian F . Telemedicine virtual reality based skin image in children's dermatology medical system. Comput Intell. 2022;38(1):229‐248. doi:10.1111/coin.12458

[11] Faheem M , Butt RA , Raza B , et al. A multiobjective, lion mating optimization inspired routing protocol for wireless body area sensor network based healthcare applications. Sensors. 2019;19(23):5072. 31757104 10.3390/s19235072 PMC6928723

[12] Ali Z , Naz S , Zaffar H , Choi J , Kim Y . An IoMT‐based melanoma lesion segmentation using conditional generative adversarial networks. Sensors. 2023;23(7):3548. doi:10.3390/s23073548 37050607 PMC10098854

[13] Sheha MA , Mabrouk MS , Sharawy A . Automatic detection of melanoma skin cancer using texture analysis. Int J Comput Appl. 2012;42(20):22‐26.

[14] Albert N , Basavesha D . An automatic helmet detection system using convolution neural network. Int J Adv Sci Innov. 2022;4(3):47741.

[15] Maurya A , Stanley RJ , Lama N , et al. A deep learning approach to detect blood vessels in basal cell carcinoma. Skin Res Technol. 2022;28(4):571‐576. doi:10.1111/srt.13150 35611797 PMC9907638

[16] Binol H , Plotner A , Sopkovich J , Kaffenberger B , Niazi MKK , Gurcan MN . Ros‐NET: a deep convolutional neural network for automatic identification of rosacea lesions. Skin Res Technol. 2020;26(3):413‐421. doi:10.1111/srt.12817 31849118

[17] Akram A , Ramzan S , Rasool A , Jaffar A , Furqan U , Javed W . Image splicing detection using discriminative robust local binary pattern and support vector machine. World J Eng. 2022;19(4):459‐466. doi:10.1108/WJE-09-2020-0456

[18] Rashid J , Ishfaq M , Ali G , et al. Skin cancer disease detection using transfer learning technique. Appl Sci. 2022;12(11):5714.

[19] Rashid J , Shabbir Qaisar B , Faheem M , Akram A , Hamid M . Mouth and oral disease classification using InceptionResNetV2 method. Multimed Tools Appl. 2023;10:1‐19.

[20] Rashid J , Khan I , Ali G , Rehman SU , Alturise F . Real‐time multiple guava leaf disease detection from a single leaf using hybrid deep learning technique. Comput Mater Contin. 2023;74(1):1235‐1257.

[21] Ullah KA , Rehman F , Anwar M , Faheem M , Riaz N . Machine learning based prediction of osteoporosis in postmenopausal women with clinical examined features: a quantitative clinical study. Health Sci Rep. 2023;11(1):1‐8. 10.1002/hsr2.1656 PMC10600334 37900094

[22] Khatibi T , Rezaei N , Ataei Fashtami L , Totonchi M . Proposing a novel unsupervised stack ensemble of deep and conventional image segmentation (SEDCIS) method for localizing vitiligo lesions in skin images. Skin Res Technol. 2021;27(2):126‐137. doi:10.1111/srt.12920 32662570

[23] Chu H , Saeed MR , Rashid J , Mehmood MT , Ahmad I . Deep learning method to detect the road cracks and potholes for smart cities. Comput Mater Contin. 2023;75(1):1863‐1881.

[24] Abhiram AP , Anzar SM , Panthakkan A . DeepSkinNet: a deep learning model for skin cancer detection. In: 2022 5th International Conference on Signal Processing and Information Security (ICSPIS) , IEEE, 2022:97‐102.

[25] Mehra A , Bhati A , Kumar A , Malhotra R . Skin cancer classification through transfer learning using ResNet‐50. In: Emerging Technologies in Data Mining and Information Security: Proceedings of IEMIS 2020 . Vol. 2. Springer; 2021:55–62.

[26] Giotis I , Molders N , Land S , Biehl M , Jonkman MF , Petkov N . MED‐NODE: A computer‐assisted melanoma diagnosis system using non‐dermoscopic images. Expert Syst Appl. 2015;42(19):6578‐6585.

[27] Taufiq MA , Hameed N , Anjum A , Hameed F . m‐Skin Doctor: a mobile enabled system for early melanoma skin cancer detection using support vector machine. In: eHealth 360°: International Summit on eHealth, , *June 14–16, 2016, Revised Selected Papers*. Springer; 2017:468‐475.

[28] Ali R , Hardie RC , De Silva MS , Kebede TM . Skin lesion segmentation and classification for ISIC 2018 by combining deep CNN and handcrafted features. *arXiv preprint arXiv:1908.05730*, 2019.

[29] Hosny KM , Kassem MA . Refined residual deep convolutional network for skin lesion classification. J Digit Imaging. 2022;35(2):258‐280. doi:10.1007/s10278-021-00552-0 35018536 PMC8921379

[30] Kassem MA , Hosny KM , Damaševičius R , Eltoukhy MM . Machine learning and deep learning methods for skin lesion classification and diagnosis: a systematic review. Diagnostics. 2021;11(8):1390. doi:10.3390/diagnostics11081390 34441324 PMC8391467

[31] Hosny KM , Kassem MA , Foaud MM . Skin melanoma classification using ROI and data augmentation with deep convolutional neural networks. Multimed Tools Appl. 2020;79(33):24029‐24055. doi:10.1007/s11042-020-09067-2

[32] Tschandl P , Rosendahl C , Kittler H . The HAM10000 dataset, a large collection of multi‐source dermatoscopic images of common pigmented skin lesions. Sci Data. 2018;5(1):1‐9. 30106392 10.1038/sdata.2018.161 PMC6091241

[33] Xie Y , Zhang J , Xia Y , Shen C . A mutual bootstrapping model for automated skin lesion segmentation and classification. IEEE Trans Med Imaging. 2020;39(7):2482‐2493. 32070946 10.1109/TMI.2020.2972964

[34] Zhuang D , Chen K , Chang JM . CS‐AF: a cost‐sensitive multi‐classifier active fusion framework for skin lesion classification. Neurocomputing. 2022;491:206–216.

[35] Zhang L , Yang G , Ye X . Automatic skin lesion segmentation by coupling deep fully convolutional networks and shallow network with textons. J Med Imaging. 2019;6(2):024001. 10.1117/1.JMI.6.2.024001 PMC6462764 31001568

[36] Khan MA , Sharif M , Akram T , Damaševičius R , Maskeliūnas R . Skin lesion segmentation and multiclass classification using deep learning features and improved moth flame optimization. Diagnostics. 2021;11(5):811. 33947117 10.3390/diagnostics11050811 PMC8145295

[37] Khamparia A , Singh PK , Rani P , Samanta D , Khanna A , Bhushan B . An internet of health things‐driven deep learning framework for detection and classification of skin cancer using transfer learning. Trans Emerg Telecommun Technol. 2021;32(7):e3963. doi:10.1002/ett.3963

[38] Xiao J , Xu H , Fang D , Cheng C , Gao H . Boosting and rectifying few‐shot learning prototype network for skin lesion classification based on the internet of medical things. Wireless Netw. 2023;29(4):1507‐1521. doi:10.1007/s11276-021-02713-z

[39] Han Y , Lei Y , Shkolnikov V , et al. An ensemble method with edge awareness for abnormally shaped nuclei segmentation. In: Proceedings of the IEEE/CVF Conference on Computer Vision and Pattern Recognition . 2023:4314‐4324.

[40] Pan Y , Liu J , Cai Y , et al. Fundus image classification using Inception V3 and ResNet‐50 for the early diagnostics of fundus diseases. Front Physiol. 2023;14:160. 10.3389/fphys.2023.1126780 PMC9975334 36875027

[41] Lee G , Yonrith P , Yeo D , Hong A . Enhancing detection performance for robotic harvesting systems through RandAugment. Eng Appl Artif Intell. 2023;123:106445.

[42] Alsahafi YS , Kassem MA , Hosny KM . Skin‐Net: a novel deep residual network for skin lesions classification using multilevel feature extraction and cross‐channel correlation with detection of outlier. J Big Data. 2023;10(1):105.

[43] Levin AA , Klimov DD , Nechunaev AA , et al. Assessment of experimental OpenCV tracking algorithms for ultrasound videos. Sci Rep. 2023;13(1):6765. 37185281 10.1038/s41598-023-30930-3 PMC10130022

[44] Cao Z , Zhu L . Segmentation algorithm of digital dorsal joint pattern image based on morphological operation. In: Eighth International Conference on Electronic Technology and Information Science (ICETIS 2023). SPIE; 2023:377‐382.

[45] Khan SA , Muneer R . A novel thresholding for prediction analytics with machine learning techniques. Int J Comput Sci Netw Secur. 2023;23(1):33.

[46] Mimura K , Nakamura K . Datasets for training and validating a deep learning‐based system to detect microfossil fish teeth from slide images. Data in Brief. 2023;47:108940. 36845646 10.1016/j.dib.2023.108940 PMC9945703

[47] Celik G . Detection of Covid‐19 and other pneumonia cases from CT and X‐ray chest images using deep learning based on feature reuse residual block and depthwise dilated convolutions neural network. Appl Soft Comput. 2023;133:109906. 36504726 10.1016/j.asoc.2022.109906 PMC9726212

[48] Wang Y , Xiao Z , Cao G . A convolutional neural network method based on Adam optimizer with power‐exponential learning rate for bearing fault diagnosis. J Vibroengineering. 2022;24(4):666–678.

[49] Jocher G , Ayush C , Alex S , et al. ultralytics/yolov5: v6. 2–yolov5 classification models, apple m1, reproducibility, clearml and deci. ai integrations. Zenodo; 2022.

[50] Xie F , Yang J , Liu J , Jiang Z , Zheng Y , Wang Y . Skin lesion segmentation using high‐resolution convolutional neural network. Comput Methods Programs Biomed. 2020;186:105241. 31837637 10.1016/j.cmpb.2019.105241

[51] Kaymak R , Kaymak C , Ucar A . Skin lesion segmentation using fully convolutional networks: a comparative experimental study. Expert Syst Appl. 2020;161:113742.

[52] Wang J , Wei L , Wang L , Zhou Q , Zhu L , Qin J . Boundary‐aware transformers for skin lesion segmentation. In: Medical Image Computing and Computer Assisted Intervention–MICCAI 2021: 24th International Conference , Strasbourg, France *, September 27–October 1, 2021, Proceedings, Part I 24*. Springer; 2021:206‐216.

[53] Liu L , Tsui YY , Mandal M . Skin lesion segmentation using deep learning with auxiliary task. J Imaging. 2021;7(4):67. 34460517 10.3390/jimaging7040067 PMC8321325

[54] Dai D , Dong C , Xu S , et al. Ms RED: a novel multi‐scale residual encoding and decoding network for skin lesion segmentation. Med Image Anal. 2022;75:102293. 34800787 10.1016/j.media.2021.102293

[55] Liu Qi , Wang J , Zuo M , et al. NCRNet: neighborhood context refinement network for skin lesion segmentation. Comput Biol Med. 2022;146:105545. 35477048 10.1016/j.compbiomed.2022.105545

[56] Shao D , Ren L , Ma L . MSF‐Net: a lightweight multi‐scale feature fusion network for skin lesion segmentation. Biomedicines. 2023;11(6):1733. 37371828 10.3390/biomedicines11061733 PMC10296632

[57] Kaur R , Ranade SK . Improving accuracy of convolutional neural network‐based skin lesion segmentation using group normalization and combined loss function. Int J Inf Technol. 2023; 15:1–9.36721525

